# Modeling Amyotrophic Lateral Sclerosis Progression: Logic in the Logit

**DOI:** 10.7759/cureus.24887

**Published:** 2022-05-10

**Authors:** Afaf Shaabi

**Affiliations:** 1 Neurological Surgery, King Faisal Medical City, Abha, SAU

**Keywords:** amyotrophic lateral sclerosis, neuro degenerative diseases, alsfrs-r, disease progression, mathematical modeling

## Abstract

Background

Amyotrophic lateral sclerosis functional rating scale-revised (ALSFRS-R) has emerged as a clinical prognostic marker for clinical and research purposes in amyotrophic lateral sclerosis (ALS). However, tools for predicting disease progression are still underdeveloped. The aim of this study was to mathematically model ALS progression to provide a reliable and personalized approach to the prognosis for ALS patients. Also, it aimed to provide a reliable prediction tool for the current and newly diagnosed patients.

Methods

Twenty patients from the South-East England Amyotrophic Lateral Sclerosis register (SEALS) database were included in the analysis. A non-linear logistic regression model was used to describe disease progression from baseline health to the theoretical maximum disease. The reliability of predicted variables and correlation between model parameters were assessed separately for each subject.

Results

The logistic regression model best described the disease progression in patients with a high progression rate. Most notably, the model fitted better when a patient has progressed enough to approximately the midpoint of the functional rating scale. The model failed to characterize the disease course in patients defined as slow progressors. Furthermore, the linear relationship between the rate of progression and time since onset at ALFRS-R score of 24 was evident in 65% of patients.

Conclusion

These results indicate that the rate of disease progression and time when ALSFRS-R declines to half the maximum score are correlated with functional outcomes. Nonetheless, the logistic model failed to describe disease course in patients with slow progression rates. Different rates of progression can be attributed to the genetic heterogeneity of ALS. Thus, clinicians and patients can benefit from adding a gene factor to the equation. With the outlined limitations, the model can provide a good prognostic tool.

## Introduction

Amyotrophic lateral sclerosis (ALS) is a fatal neurodegenerative disease [1]. Most reported cases are sporadic, but the analysis of the extended family has shown that up to 20% of ALS patients have a family history of ALS [2]. The etiology of ALS is complex, with a growing number of underlying genetic and environmental factors [3]. To date, several mutations in culprit genes have been linked to both familial and sporadic forms of ALS, such as fused in sarcoma gene (FUS), C9orf72, superoxide dismutase 1 (SOD1), and TAR DNA-binding protein (TARBDP) [4]

This genetically heterogeneous syndrome is characterized by a progressive loss of motor neurons in the brain and spinal cord leading to a gradual and irreversible paralysis. Although it is often referred to as motor neuron disease (MND), ALS patients also exhibit non-motor cognitive and behavioral manifestations [5]. Frontotemporal dementia and mild cognitive impairment are common comorbidities [5]. In a study conducted by McLaughlin et al., ALS has been genetically linked to schizophrenia, suggesting a common etiology [6].

Unfortunately, as the disease progresses, respiratory function deteriorates due to a weakening chest wall, which then warrants assisted ventilation [7]. Death is usually eminent three years after the onset of symptoms [7]. In Europe, the prevalence is 2·6-3·0 cases per 100 000 people [8,9]. In fact, the reported incident rate is variable in different population-based studies: it has been estimated as 1·75 and 2·16 cases per 100 000 people worldwide [10] and of European descent, respectively [9]. The incident rate is slightly higher among men than women (3·0:2·4) [9]. Also, the age of ALS disease onset was 61·8 ± 3·8 years (mean ± SD) worldwide [9,11] while the median interquartile range age at diagnosis in Europe was 65·2 (56·0, 72·2) years [9]. ALS is a diagnosis of exclusion based on the revised El Escorial criteria, which are mainly used for patient recruitment in ALS research [12]. No confirmatory test exists, and the average duration between symptoms onset and diagnosis is 11·5 months [13]. Probably, this delay is due to the insidious nature of the disease, focal onset, and non-linearity of its progression. There is no cure for ALS but the battle against this disease is promising. Riluzole, for example, is the standard of care in most countries and has been shown to increase the median survival by two to three months [14].

ALS functional rating scale (ALSFRS)

A functional rating scale for ALS was developed to clinically monitor the degree of patient disability over time [15]. Since then, it has become the most widely used outcome measure by clinicians and researchers [16]. It is a questionnaire-based scale that covers four domains: bulbar, gross and fine motor, and respiratory functions [16]. A scale from zero (maximum disability) to 48 (normal) is set, and a functional decline is defined as a decreasing score over time [16]. Since 1999, a revised form has been adopted (ALFRS-R) that incorporates more items to the respiratory function [15].

Modeling disease progression

In the transition from full health to a maximum disability, ALS patients deteriorate in a non-linear fashion [17]. It has been shown that the observed rate of decline plays a significant role in predicting functional and survival outcomes [11,18], This interesting finding urged researchers to investigate the exact shape of the progression curve [19]. Poesen et al. argue that disability does not abruptly worsen as reflected by ALSFRS-R scores after symptoms onset but declines slowly at first [19]. Then, a phase of constant progression follows until the severity of disability attains a more gradual decline [19]. According to this observation, a sigmoidal prediction model described ALS progression best [19]. It was able to characterize the disease course in two parameters that can work independently: the time in months when the ALSFRS-R score reaches 24 and the slope of the curve at that point [19]. A reliable prediction model will not only utilize ALSFRS-R scores of current and future patients but also benefit from the stored data of deceased ALS patients.

The objective of this study was to mathematically model ALS progression to provide a reliable and personalized approach to prognosis for current and newly diagnosed patients. Drawing upon the hypothesis of Poesen et al., [19] we adopted their logistic model. The model is based on the ALS functional rating scale-revised (ALSFRS-R) and the slope of progression which provided valuable insights into the disease progression [18].

In summary, ALS is a fatal syndrome of multifactorial etiology. Currently, there is no available disease-modifying treatment. Most importantly, ALS patients progress at variable rates, which indicates the need to develop an individualized prediction tool to guide therapeutic endeavors. This article was previously posted to the the Lancet preprint server on March 01, 2022 [20]. It is also a concise version of the dissertation submitted to King's College London in July 2017.

## Materials and methods

Study subjects

This is a cohort of 20 patients from the South-East England Amyotrophic Lateral Sclerosis register (SEALS). It is a population-based database, details of the register are described elsewhere [21]. The period of capture runs from January 01, 1993 to February 16, 2017. The study was approved by the ethics committees of the relevant institutions. Patients with at least three successive total ALSFRS-R scores were included (n= 20) with an average number of 4·5± 2·2 (mean ± standard deviation (SD)) observations, first ALSFRS-R score of 40·8 + 4·7, and last score of 25·15+ 7·9. The average age of onset was 59·3 ± 13·49 (mean ± SD). Detailed demographics can be found in Table 1.

**Table 1:** Demographics of the cohort (n = 20).

Age of onset	Gender	Site of Onset %	ALSFRS-R scores/patient
59·3 ± 13·49 SD	45% Male (n = 9)	30% Bulbar (n = 6)	Minimum: 3
(27·43–78·69)	55% Female (n = 11)	35% Lower Limb (n = 7)	Maximum: 11 Mean: 4·5 ± 2·2 SD
		25% Upper Limb (n = 5)	
		10% Unknown (n = 2)	

Statistical analysis

The dependent variable (total ALSFRS-R scores) was obtained from the sum of the sub-scores of the functional rating scale using the sum function in Microsoft® Excel for Mac (2016) version 15.24. None of the subs-scores was missing. The independent variable (time since onset) was calculated from the dates since symptoms onset to the time of the assessment in months. The months since symptoms onset and their corresponding total ALSFRS-R scores were rearranged in increasing chronological order for each patient to signify disease progression.

Disease progression model

The sigmoidal logistic regression model was selected because, previously, it was successfully applied to describe disease course in a majority of ALS patients [19] with the following equation:

$y= \frac{y_{max}}{1+\varrho ^{\frac{\left ( \chi -D50 \right )}{d\chi }}}$

In the equation, $y$ is the total ALSFRS-R score of a patient at a given time; $Y_{max}$ is the theoretical maximum ALSFRS-R score of 48; $x$ is the time in months since symptoms onset to the date of $Y$ estimation; $D50$ is the time in months when the patient reaches half $Y_{max}=24$; $dx$ is the slope at an ALSFRS-R score of 24. The model parameters $dx$ ​​and $D50$ are unknown and are estimated from the conventional slope equation from a minimum set of two ALSFRS-R.

Hypothesis testing

To test whether a logistic regression model given by the model equation is a good tool to describe ALS progression, a non-linear regression analysis was run by the statistics software, SPSS version 23 (IBM Corp., Armonk, NY). The model parameters $Y_{max}$, $D50$ and $dx$ were substituted in the model equation as initial values were derived from the conventional slope equations. Model stability was assessed using non-linear regression analysis with bootstrapping as an internal model evaluation technique. The optimal model parameter estimates were compared to a preset confidence interval of 95%. The predictive model performance was evaluated using reliability analysis to ascertain the absolute agreement between the observed ALSFRS-R scores and predictions generated by the model.

## Results

Estimation of model parameters

For the cohort of 20 patients in our database, we have determined $D50$ and $dx$ in 65% of patients (n = 13) using the non-linear regression analysis with initial values derived from the conventional estimation of ALSFRS-R progression shown in Table 2.

**Table 2:** Descriptive statistics of initial and optimal values of the model parameters. $dx$ is the rate of progression, and $D50$ is the time since symptoms onset in months at ALSFRS-R =24. SD: standard deviation.

	N	Minimum	Maximum	Mean	SD
Initial dx	13	0·70	8·00	2·2002	1·87686
Optimal dx	13	1·26	17·18	6·4615	3·84198
Initial D50	13	14·11	54·14	30·9701	12·74267
Optimal D50	13	13·90	51·72	29·5540	11·95293

Model evaluations in 35% of patients (n = 7) could not find a better fit of the model iteratively and the simulation stopped after 0 evaluations. The software reported that optimal solutions of $dx$ and $D50$ were equal to the initial values derived from the conventional slope equation as shown in Table 3.

**Table 3:** Descriptive statistics of initial values of the model parameters. The initial values were equal to the optimal values estimated. Dx is the rate of progression, and D50 is the time since symptoms onset in months at ALSFRS-R =24. SD: standard deviation.

	N	Minimum	Maximum	Mean	SD
dx	7	0·08	0·40	0·2117	0·11187
D50	7	59·14	282·00	135·9184	73·11256

Hypothesis testing

**Figure 1:**
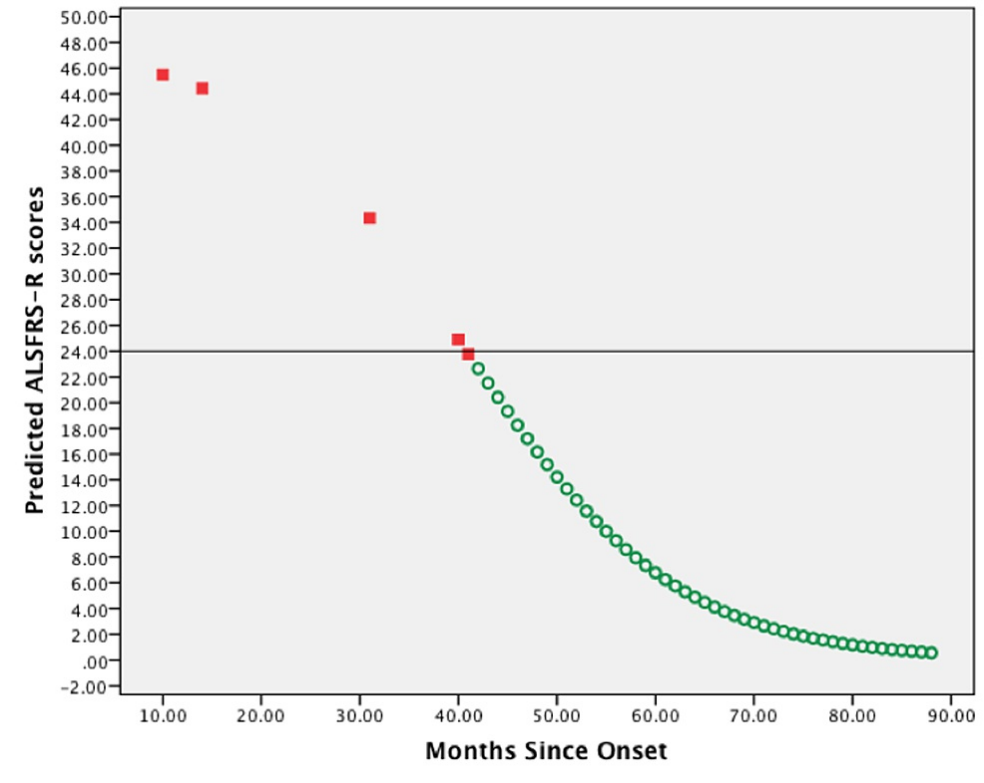
Scatterplot of predicted ALSFRS-R scores vs. months since symptoms onset of one representative patient. The first five predicted ALSFRS-R scores were plotted against months since symptoms onset of the documented patient's visits (red squares). Predicted ALSFRS-R scores were also plotted against months since symptoms onset of increments of =1 (green circles). A horizontal line is shown at the inflection point (ALFRS-R score = 24) of the sigmoidal curve. The predictions were generated using optimal parameter values by iteration. ALSFRS-R: amyotrophic lateral sclerosis functional rating scale-revised.

The graphic representation in Figure 1 demonstrates predicted ALSFRS-R scores generated by model evaluations using optimal parameter estimates solved by iteration. Here the initial value for the slope was 1·076, and by iteration, it was 10·641. Additionally, the initial value for $D50$ was 46·505, and iteratively, it was 40·796. Noticeably, for 65% of patients (n = 13) 65% whose parameter estimates were found iteratively, similar sigmoidal curves were produced.

**Figure 2:**
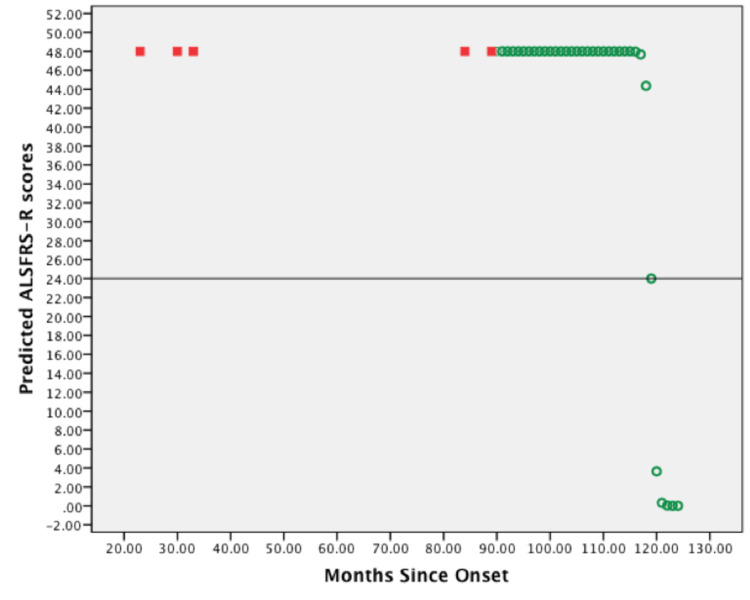
Scatterplot of predicted ALSFRS-R scores vs. months since symptoms onset of one representative patient. First five predicted ALSFRS-R scores were plotted against months since symptoms onset of the documented patient's visits (red squares). Predicted ALSFRS-R scores were also plotted against months since symptoms onset of increments of = 1 (green circles). A horizontal line is shown at the inflection point (ALFRS-R score = 24) of the sigmoidal curve. The predictions were generated using initial parameter values. ALSFRS-R: amyotrophic lateral sclerosis functional rating scale-revised.

The graphic representation in Figure 2 demonstrates predicted ALSFRS-R scores generated by model evaluations. Here, the initial values for the slope and D50 were 0·4 and 119, respectively. Noticeably, for 35% of patients (n = 7), whose parameter estimates were found iteratively sigmoidal curves with a similar shape were produced, in which the predicted ALSFRS-R scores have equal values of 48 repetitively at first, and then sharply decrease from 48 to 24 and zero scores. In this case, the inflection point is perpendicular to both ends of the curve.

**Figure 3:**
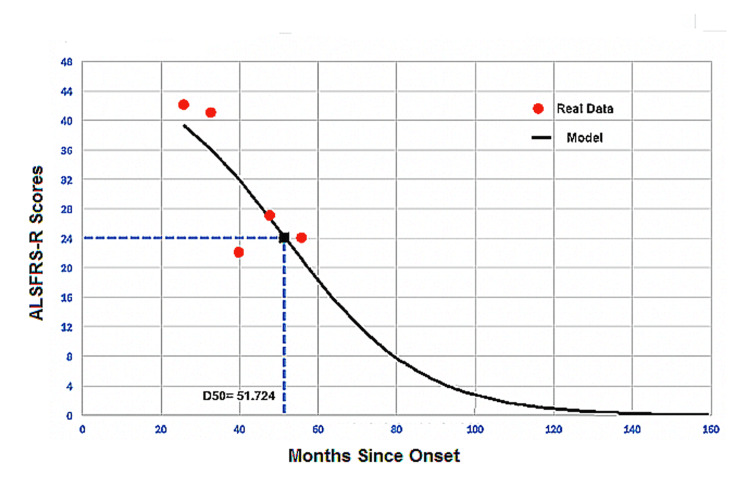
Simulated amyotrophic lateral sclerosis (ALS) disease curve. Real data (red dots) the model curve fitted by the simulation model (continuous black line) of one representative ALS patient.

The scatterplot in Figure 3 shows the real ALSFRS-R scores (red dots) fitted to a sigmoidal curve model (continuous black line). Here, the ALSFRS-R score reaches 0·9 and 0·0 at 118 and 159 months, respectively. The estimate is 51·724 months. The black square represents the point when the patient reaches half the maximum ALSFRS-R score i.e., for ALSFRS-R score of 24, the slope estimate here is 17·184. Notice the model performance when this patient gained back five ALSFRS-R scores at 48 months from ALSFRS-R of 22 at month no. #40.

**Figure 4:**
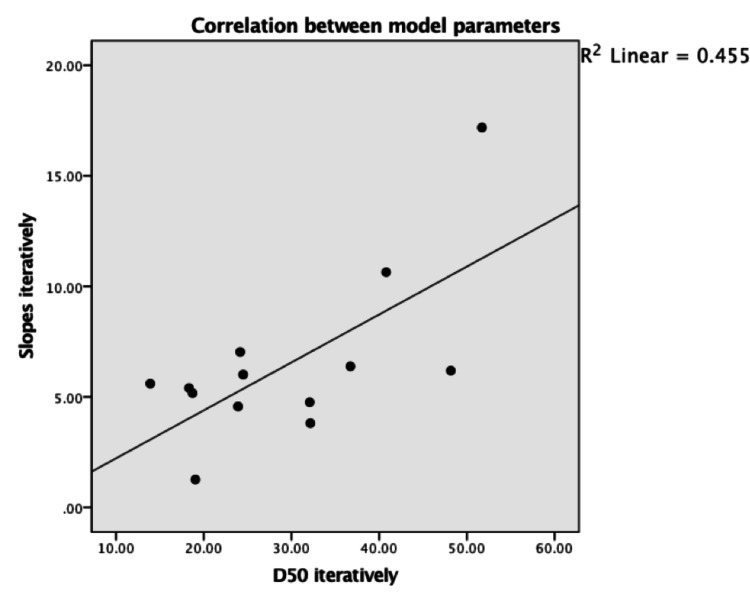
Relationship between slopes and D50 in 65% (n = 13) of patients.

Scatterplot with the best fit line in Figure 4 describes the intermediate linear relationship (R squared = 0.455) between model parameters (slope = $dx$ and $D50$). The confidence interval is 95% from bootstrap percentiles.

**Figure 5:**
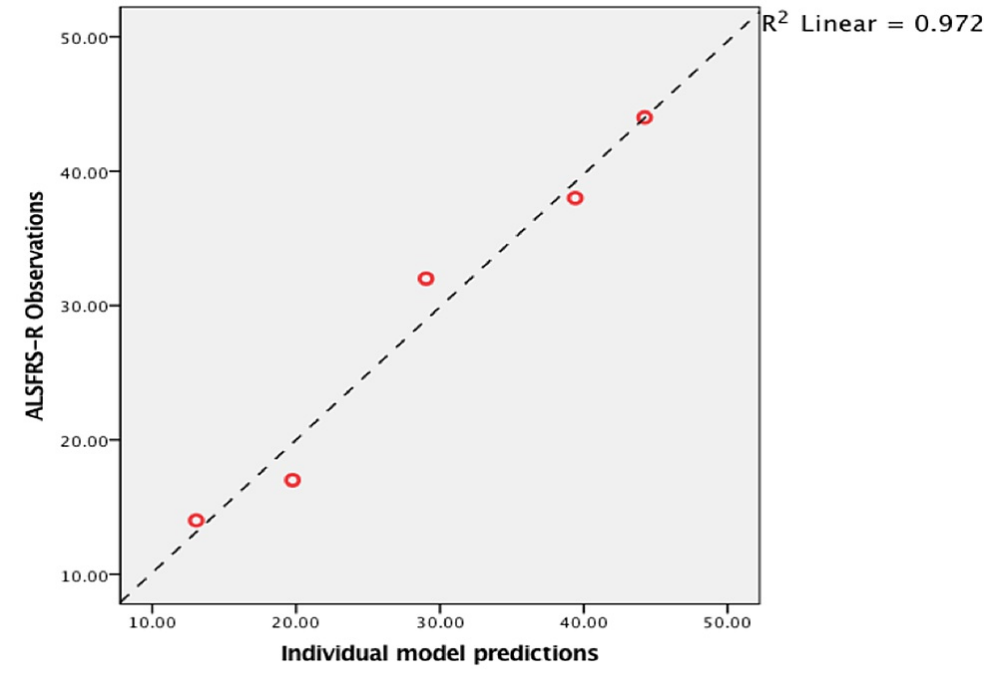
Relationship between observed vs. predicted ALSFRS-R scores of a representative ALS patient. ALSFRS-R: amyotrophic lateral sclerosis functional rating scale-revised, ALS: amyotrophic lateral sclerosis.

Scatterplot with the best fit line in Figure 5 demonstrates a strong linear relationship (R squared = 0·972) between the observed ALSFRS-R scores and predicted ALSFRS-R scores estimated by the model. P-value < 0·001, 95%, confidence interval (0·958-0·999), Cronbach's alpha 0·993.

**Figure 6:**
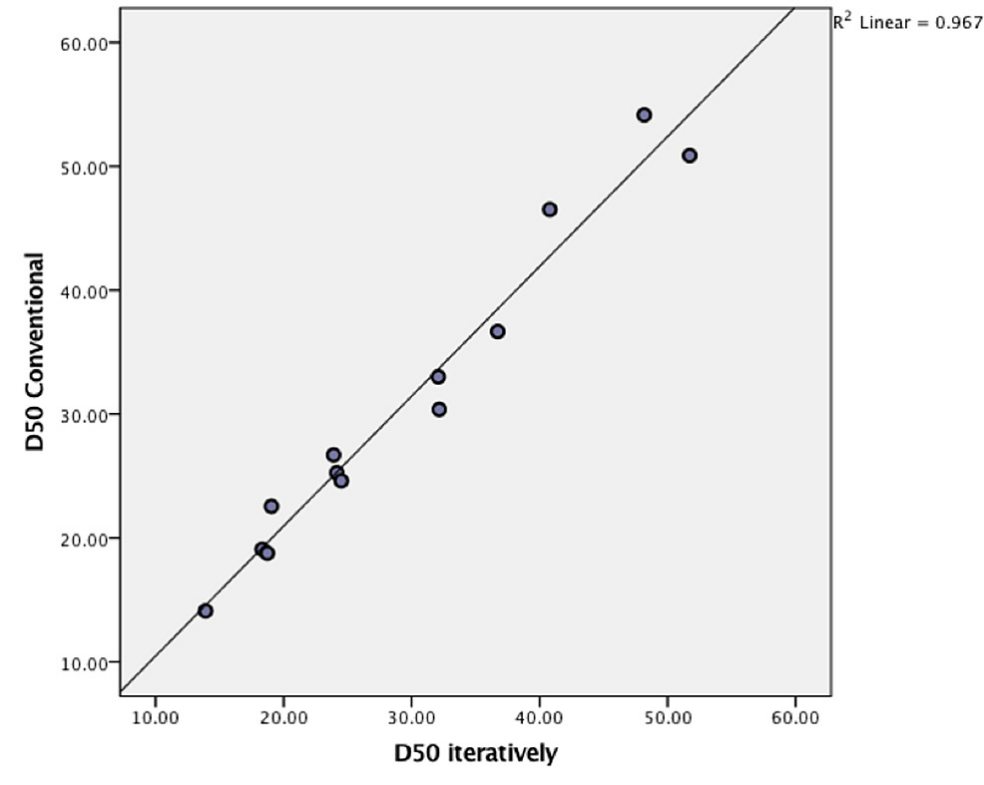
Relationship between values of conventional D50 (y axis) vs. optimal values estimated iteratively (x axis).

Scatterplot with best fit line in Figure 6 shows strong linear relationship (R squared = 0.967) between calculated $D50$ using conventional slope equation and the optimal solutions generated by the model in 65% of patients (n = 13). P-value < 0·001, 95% confidence interval (0·962-0·996), Cronbach's alpha 0·991.

## Discussion

The results of the analyses show that the logistic regression model provided an accurate description of the course of ALSFRS-R score decline p-value < 0·001 (Figure 5). It proves that the ALSFRS-R score decays in a curvilinear fashion rather than linearly. These results are consistent with what has been reported by Gordon et al. and Poesen et al. [17,19]. Furthermore, Gomeni and Fava confirmed that using a linear model based on ALSFRS-R scores produced significant bias when describing disease course as compared to a non-linear model [8]. However, we found that the model describes the disease course best in 65% of cases, while Poesen et al. reported this percentage as high as 98% [19]. This discrepancy is probably due to the small sample size analyzed in our study and using different techniques in implementing the iterative method and different statistical software. Interestingly, the model could not estimate optimal solutions for parameters, i.e. $D50=$ time in months at ALSFRS-R score of 24 and $dx$ slope at that point, iteratively in 35% of patients (Figure 2). These patients had slower progression rates than the rest, with an average of 0·2117 scores/month (0·08-0·40). No further analysis was used to define this rate as slow, but it is consistent with Poesen et al.’s definition of slow progressors ($dx$ < 0·365) [19]. Table 2 outlines the details of parameter values in this population.

These findings are in line with what has been reported by Labra et al, where three groups with different progression rates were identified [22]. Most noticeably, a group with a median progression rate of < 0·47 was identified with a slow functional decline with a lower risk of death compared to a group with a faster progression rate of > 1·11. Based on the recently published studies and our findings, the prognostic value of the slope appears to be identical in different ALS cohorts. We suggest that a reliable cut-off value of progression speed should be developed. This important step can aid clinicians in the proper identification of patients with a more aggressive disease course and earlier introduction of treatment and rehabilitation.

The model was not successful in describing the ALS disease course in slowly progressing patients. This is probably because a smaller slope = $dx$ means a value closer to zero and positive figures, which converts the function of decay toward growth in the logistic model. This phenomenon was ascertained by running the simulation without constraining the numerator $Y_{max}$ in the model equation between (0,48). The predicted values of ALSFRS-R scores were > 48 and < zero despite inserting 48 as the initial value for the numerator. Unlike findings reported by Poesen et al, the model failed to describe the disease course in 35% of patients compared to 2% in their study [19]. Moreover, in Figure 4, we found that the model parameters were moderately linear (R squared = 0·455). Meanwhile, Poesen et al. showed strong linearity (R squared= 0·932) [19]. Therefore, neither can serve as an independent prognostic factor to describe the decay in ALSFRS-R sufficiently.

The Weibull model in a study by Gomeni and Fava categorized ALS patients in a bivariate distribution (slow and fast) according to the rate of decline from the baseline ALSFRS-R score [18]. They were able to identify two sub-groups within their sample with a significant comparable probability of a patient belonging to a certain cluster [18]. The robustness of the predicted ALSFRS-R scores also support our results about the two categories that emerged as slow and fast progressors. In our model, we constrained the ALSFRS-R to the maximum of 48 as the highest possible functional outcome at disease onset. This value produced falsely repeated predictions of 48 in patients with a slow progression rate. But, in the study by Gomeni and Fava, the baseline ALSFRS-R score was used to estimate accurate predictions in both clusters [18]. This, perhaps, suggests a more prognostic significance to the first observed ALSFRS-R score rather than the theoretical maximum. The prognostic potential for the initial ALSFRS-R score has also been validated by Labra et al. [22].

The calculation of the initial values of $D50$, indicated that the ALSFRS-R set, from which the slope of the midpoint is derived, should be sufficiently less than 48 to give a better estimation. Apparently, an ALSFRS-R set from the second one-third of the scale between 0 to 48, produced a better approximation to the midpoint. This method suggests that for the model to fit accurately, the patient must progress markedly enough from the baseline. Obviously, the logistic model has defined added constraints to the inclusion criteria as necessary for a successful application of this methodology. In fact, this limits the recruitment process to a certain population of ALS patients.

According to the results of the $D50$ estimation in Figure 6, strong linearity is evident (R squared= 0·967) between the iterative and the conventional estimation of the parameter. On the other hand, $dx$ estimates could not show that same relationship. This might indicate that the midpoint of the ALSFRS-R strongly correlates with the disease course, which warrants more investigation on this point.

Expectedly, the fitted model curve in Figure 3 depicted a sigmoid-shaped curve with defined downward steepness. This behavior is based on the decay function of the slope coefficient. It assumes that ALS patients constantly deteriorate and lose their ALSFRS-R scores at each point in time throughout the disease course. Thus, when a patient's condition improved probably due to therapeutic interventions [18], the model did not behave accordingly. For example, in Figure 3, the patient gained back 5 ALSFRS-R scores at 48 months from ALSFRS-R of 22 at 40 months. However, the model predicted ALSFRS-R scores of 31 and 26. This phenomenon may also explain the reason why the model describes fast progressors best rather than slow progressors. A patient with a slow deterioration rate is represented in Figure 2, where $dx$​​​​​​​ is 0·4. Because this is a novel approach suggested by Poesen et al. [19], we did not encounter similarly shaped curves for slow progressors.

## Conclusions

In summary, with the relevant limitations, we tested the performance of a logistic regression model in describing the disease course in ALS patients of different progression rates. We found that the ALS disease progression model can describe the disease course in a limited number of patients. These results indicate that the rate of disease progression $dx$ and time when ALSFRS-R declines to half the maximum score $D50$ are correlated with functional outcomes reflected by ALSFRS-R scores. Further, these results suggest that the logistic model failed to describe disease course in patients with slow progression rates. Thus, clinicians and patients can benefit from adding a gene factor to the equation that could address the role of heterogeneity in ALS. One must determine the extent to which an individual gene mutation contributes to the disability first. In addition to other prognostic factors, such as gender, site of disease onset, age, and therapeutic interventions, a sub-score analysis for the ALSFRS-R components might offer additional clarity to determine the weight of each functional aspect on the overall prognosis in an independent cohort with large sample size.
